# Effects of Acute and Chronic Heavy Metal (Cu, Cd, and Zn) Exposure on Sea Cucumbers (*Apostichopus japonicus*)

**DOI:** 10.1155/2016/4532697

**Published:** 2016-06-12

**Authors:** Li Li, Xiangli Tian, Xiao Yu, Shuanglin Dong

**Affiliations:** The Key Laboratory of Mariculture, Ministry of Education, Fisheries College, Ocean University of China, Qingdao 266003, China

## Abstract

Acute and chronic toxicity tests were conducted with sea cucumber (*Apostichopus japonicus*) exposed to heavy metals. Acute toxicity values (96 h LC50) were 2.697, 0.133, and 1.574 mg L^−1^ for Zn, Cu, and Cd, respectively, and were ranked in order of toxicity: Cu > Cd > Zn. Under chronic metal exposure the specific growth rates of sea cucumbers decreased with the increase of metal concentration for all the three metals. After acute metal exposure, the oxygen consumption rate (OCR) decreased. The OCRs in all groups were significantly different than control (*P* < 0.05) except in the group treated with 1.00 mg L^−1^ Zn (*P* < 0.05), where the increase of OCR was observed. The OCRs in groups chronically exposed to metals were significantly lower than that in the control group (*P* < 0.05). The activity of both pyruvate kinase (PK) and hexokinase (HK) in sea cucumbers followed: respiratory tree > muscle > intestine in natural sea water. After chronic Zn, Cu, and Cd exposure, the change pattern of HK and PK in respiratory tree, muscle, and intestine varied slightly. However, the activity of the enzyme showed a general trend of increase and then decrease and the higher the exposure concentration was, the earlier the highest point of enzyme activity was obtained.

## 1. Introduction

The sea cucumber* Apostichopus japonicus* is a dominant mariculture species in northern coastal areas in China with the production of 194,000 t in 2013 [1, 2]. However, with the rapid development of intensive farming and industry activities, more liquid effluents with high levels of heavy metals have been discharged into the environment, which posed a potential threat to sea cucumber culture [3]. In general, heavy metals could be divided into different categories according to their toxicity and function. Metals such as cadmium (Cd) and lead (Pb) are biologically nonessential and their toxicities rise with increasing concentrations. Metals such as copper (Cu), zinc (Zn), and iron (Fe) are essential elements playing important roles in biological systems. However, the essential elements can also be detrimental to living organism at high concentrations [4].

Heavy metal exposure was considered to be associated with fish deformities and has been a subject of concern for many decades [4]. Meanwhile, studies have shown that exposure to heavy metals in aquatic environment could change the metabolic activities and many other physiological characteristics in crustaceans [5]. As fundamental physiological activities of animal energy metabolism, respiration is directly associated with the amount of energy released from the oxidation of food substratum. Therefore, it is a good indicator to evaluate the toxicant effects caused by heavy metals [5, 6]. For example, oxygen consumption rate (OCR) was used to study the adverse effect of heavy metal exposure on Pacific white shrimp, Green-lipped mussel, and ridgetail white prawn [6–8]. Both pyruvate kinase (PK) and hexokinase (HK) are key enzymes of glycolysis. The alteration of activity of these enzymes could change the metabolic level of the animal. Several environmental factors such as salinity, temperature, and diet can influence the glucose metabolism in aquatic animals by altering the enzyme activities [2, 9].

Although the effect of heavy metal exposure on crustaceans and molluscan has been extensively studied, there is not much information available as to what happens in sea cucumber. In the present study, we evaluated the effect of one commonly found nonessential element (Cd) and two essential elements (Zn and Cu) on the survival, specific growth rate (SGR), OCR, and activity of metabolic enzymes in sea cucumber. This study provides a reference of the safety concentration of heavy metals in sea cucumber culture and most importantly provides basic data about the toxicity mechanism of sea cucumber to heavy metal exposure.

## 2. Materials and Methods

### 2.1. Collection and Maintenance of Animals

Juvenile sea cucumbers (*Apostichopus japonicus*) around 15 g were purchased from a local farm in Jiaonan district and transported to the laboratory located on the campus of Ocean University of China (Qingdao, Shandong, China). The animals were acclimated for 10 days in nature seawater continuously aerated with air stones. One-half or one-third of the rearing water was exchanged by fresh equitemperature seawater every day to ensure high water quality. The sea cucumbers were fed ad libitum every day at 08:00 on formulated feed (crude protein ≥ 23.0%, 3.0–5.0% fat, ash ≤ 18%, fiber ≤ 8.0%, and moisture ≤ 11.0%). After acclimation, the healthy individuals with an average weight of 15.42 ± 2.07 g were selected for the toxicity study.

The metal salts ZnSO_4_·7H_2_O, CuSO_4_·5H_2_O, and CdCl_2_·2.5H_2_O were dissolved in deionized water to prepare stock metal solution and stored at 4°C. Seawater used in the experiment was precipitated and filtered by a composite sand filter. The temperature, salinity, and pH of the seawater used during the experiment were controlled at 17.2 ± 0.2°C, 29.0 ± 2.0‰, and 7.87 ± 0.29, respectively. The ammonia and nitrite concentrations were kept at less than 0.011 and 0.0026 mg L^−1^, respectively.

### 2.2. Acute Toxicity Study

A preliminary experiment was conducted to determine the highest concentrations of Zn, Cu, and Cd, respectively, causing no mortality and the lowest concentrations of Zn, Cu, and Cd, respectively, causing 100% mortality of sea cucumber in 96 h. Concentrations of the treatments were set up based on the equal logarithm intervals method with nature seawater as control (Table 1). Using Zn as an example, the highest concentration of Zn causing no mortality was 1 mg L^−1^ and the low lowest concentration of Zn causing 100% mortality was 6 mg L^−1^. The logarithms of 1 and 6 to base 10 were 0 and 0.78, respectively. The interval between 0 and 0.78 was divided into five equal parts with six numbers. Using the number 10 as the base and the six corresponding numbers as exponent, the concentrations of treatments were set up as Table 1. The study was conducted in glass aquariums (53 cm × 28 cm × 34 cm) with five sea cucumbers in each aquarium. Five replicates were conducted for each treatment. The sea cucumbers were not fed two days before the study to empty intestine. The culture water was 100% changed every 24 h. The activities of the experimental animals were continuously observed and the dead individuals were picked out. The number of death was recorded at 24 h, 48 h, 72 h, and 96 h.

The LC50 is the concentration of toxicant causing 50% mortality of the test animals. The probit analysis (PB) method, the linear regression of probit mortality on log dosage, was employed to obtain a regression equation to estimate the 24 h, 48 h, 72 h, and 96 h LC50 [10]. The maximum allowable toxicant concentration (MATC) is the concentration of toxicant that may be present without causing harm. It can be calculated by multiplying the 96 h LC50 by application factor (AF). An AF of 0.05 is suggested by Boyd for general use [11].

### 2.3. Chronic Toxicity Study

#### 2.3.1. Experimental Design and Management

A 15 d chronic toxicity test was conducted in glass aquariums (53 cm × 28 cm × 34 cm). Concentrations of Zn, Cu, and Cd were set up as 1/200, 1/100, 1/50, and 1/10 of 24 h LC50 value of each element with nature sea water as control (Table 2). Ten replicates were conducted for each treatment. Sea cucumbers were randomly picked up in five of ten replicates and assigned to samples for HK and PK analysis. Muscle, intestine, and respiratory tree samples were collected on 0 h, 12 h, 24 h, 5 d, 10 d, and 15 d during the experiment. Three animals were randomly sampled from five aquariums. Muscle was removed from the posterior of the body. The whole intestine was removed by an incision at the esophagus and cloaca. It was then cut longitudinally and washed thoroughly in ice-cold distilled water. After rinsing, the three tissues were dried with filter paper, and each sample was frozen with liquid nitrogen in an Eppendorf tube (1.5 mL) and stored at −80°C until analysis.

Metal concentration in the test solution used in the acute and chronic toxicity study was measured using the inductively coupled plasma-optical emission spectrophotometer (ICP-OES; VISTA-MPX, VARIAN). The nominal and measured concentrations of metals were listed in Table 3.

#### 2.3.2. Enzyme Activity Determination

A 0.1–0.3 g of each of the three tissues was homogenized and the ice-cold saline of quadrupled volume of the tissue was added to each sample to make a 20% homogenate. The homogenates were immediately centrifuged for 10 min at 4°C and 2000 r/min. The protein concentrations of the samples were determined with Folin phenol reagent [12]. Collected supernatants were used for the determination of activities of PK and HK using a commercial kit (Nanjing Jiancheng Bioengineering Institute, China).

The activity of PK was determined by continuously monitoring the decrease in absorbance at 340 nm using NADH-linked methods [13]. PK activities were calculated using the molar extinction coefficient of NADH (6.22 mmol^−1^ cm^−1^). The HK activity was determined by reading the absorbance values using spectrophotometer at 340 nm [14]. All enzyme activities were expressed as U mg^−1^ (unit per milligram protein), where U was defined as the enzyme causing the conversion of 1 *μ*mol of substrate min^−1^ [15].

#### 2.3.3. Specific Growth Rate

Sea cucumbers in five aquariums for each treatment were assigned to measure the growth rate of animals. The wet weight of the sea cucumbers before and after the experiment was recorded to calculate the specific growth rate (SGR) of the animal using the following equation. Before weighing, the animals were fasted for 24 h to evacuate their guts:

(1) Specific  growth  rate  SGR=ln⁡W2−ln⁡W1t2−t1×100%,

where *W*_1_ and *W*_2_ were the weights at times *t*_1_ and *t*_2_, respectively, with *t*_1_ and *t*_2_ being the first and final day of the experiment, respectively.

### 2.4. Oxygen Consumption Rate Determination

The oxygen consumption rates were measured for the sea cucumbers after 96 h acute toxicity and 15 d chronic toxicity test at all the treatments and control except the CA5 and CA6 groups (Table 1) according to the methods described by Dong et al. [16]. The two groups were not measured because of the high mortality of the animals after 96 h acute metal exposure. Briefly, an individual sea cucumber was put into a 3-L conical flask. Four replicates in each treatment and one blank control to correct for the respiration of bacteria in the water were set up. When it became quiescent after 12 h, change in oxygen content was determined before and after the test over 24 h using the Winkler method [17].

The oxygen consumption rate (*R*_0_) of sea cucumber was calculated from the following equation [18]:

(2) $$\begin{array}{c} R_{0}\left(\;mg\;O_{2}*g^{-1}*h^{-1}\;\right)=\frac{\left(C_{0}-C_{t}\right)V}{WT}, \end{array}$$

where *C*_*t*_ and *C*_0_ are the change in oxygen content (mg O_2_ L^−1^) before and after test in the test bottles and blank bottles, respectively; *V* is the volume of the bottle (L); *W* and *T* are the wet weight of sea cucumbers (g) and time of duration (h), respectively.

### 2.5. Statistical Analysis

Statistical analyses were performed using SPSS (version 17.0). The comparisons of weight, specific growth rate, and oxygen consumption rate among treatments were done by one-way ANOVA, followed by Duncan's multiple comparison tests if significant difference was reported by ANOVA. All the data were expressed as mean ± standard error.

## 3. Results

### 3.1. Acute Toxicity of Zn, Cu, and Cd on Survival and Behavior of Sea Cucumber and the LC50 Values

Effects of acute toxicity of the three metals (Zn, Cu, and Cd) on survival rate of sea cucumber are listed in Table 4. With the increase of concentration and time duration of metal exposure, the survival rate of sea cucumber decreased. The sea cucumber showed similar toxicity symptom under the stress of the three metals. In the low concentration groups, that is, the concentration with Zn of 2.05 mg L^−1^, Cu of 0.08 mg L^−1^, and Cd of 1.15 mg L^−1^, the activities of the sea cucumbers were similar to the animal in the control group in the first 24 h. The sea cucumber was absorbed on the wall or bottom of the aquarium. However, with the extension of exposure time, the absorption capacity of ambulacral foot weakened and some of the animals dropped on to the bottom of the aquarium accompanied by twisting and contraction of the body. Few individuals spontaneously rejected internal organs, that is, evisceration, followed by disappearance of spines on the body and after evisceration the individuals started to rot. The sea cucumbers were more sensitive to high concentration (Zn of 6.00 mg L^−1^, Cu of 0.20 mg L^−1^, and Cd of 2.00 mg L^−1^) metal exposure. Immediately after exposure, some individuals dropped on the bottom of the aquarium, twisted, and contracted the body followed by evisceration and death.

As exhibited in Table 5, with the extension of exposure time, the LC50 for the three metals all decreased demonstrating that the toxicity of the three metals to sea cucumber increased as the exposure time increased. The 96 h LC50 values for Zn, Cu, and Cd were 2.679, 0.133, and 1.574 mg L^−1^, respectively, while the maximum allowable toxicant concentrations (MATC) for the three metals (Zn, Cu, and Cd) were 0.135, 0.007, and 0.079 mg L^−1^, respectively.

### 3.2. Effects of Chronic Toxicity of Metals on Survival and Growth of Sea Cucumber

The survival rate and SGR of sea cucumber after chronic metal exposure are listed in Table 6. With the increase of metal concentration, the SGR of sea cucumber decreased for all the three metals. The SGRs in groups treated with Zn and Cu were significantly lower than that in control (*P* < 0.05). When the Cd concentration was over 0.044 mg L^−1^, the SGRs of sea cucumbers were significantly lower than those in control and the low concentration (0.022 mg L^−1^) treatments. Apparent uneaten feeds were found on the 7th d with Zn at the concentration of 0.150 mg L^−1^, the 10th d with Cu at the concentration of 0.010 mg L^−1^, and 12th d with Cd at the concentration of 0.088 mg L^−1^. After that, all the sea cucumbers stopped eating and began to contract their bodies into balls. Negative SGRs were recorded in treatments with the Zn, Cu, and Cd concentrations of 0.770, 0.050, and 0.440 mg L^−1^ and their survival rates were 58.3%, 75.0%, and 83.3%, respectively.

### 3.3. Effects of Metal Stress on OCR and Metabolic Enzymes of Sea Cucumber

#### 3.3.1. Acute Zn, Cu, and Cd Stress on OCR of Sea Cucumber

**Figure 1:**
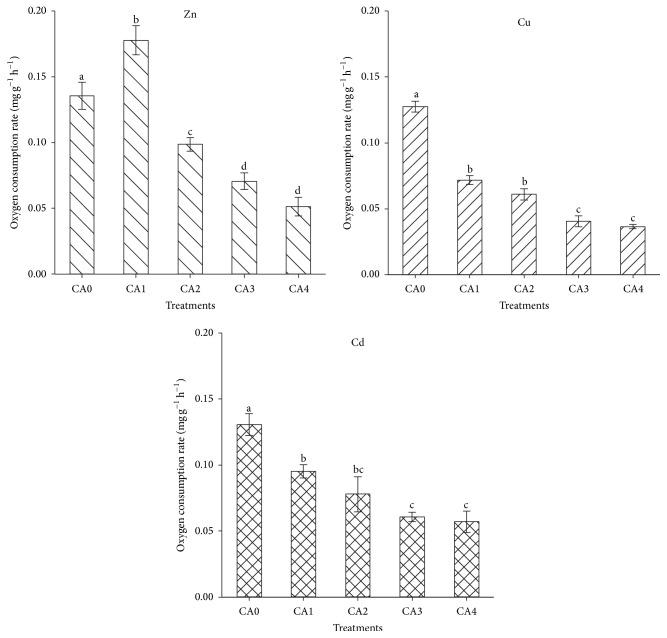
Levels of oxygen consumption rate of* Apostichopus japonicus* after 96 h acute exposure to various Zn, Cu, and Cd concentrations. The bars are the respective standard deviations (*n* = 3), and different letters above the bars indicate significant differences (*P* < 0.05).

Significant differences were found on OCR between sea cucumbers acutely exposed to different concentrations of Zn, Cu, Cd, and control (*P* < 0.05). The OCR increased significantly in the group treated with 1.00 mg L^−1^ Zn (*P* < 0.05), while in the other groups, similar trend was observed: with the increase of metal concentrations, the OCRs decreased and were significantly lower than that in control (*P* < 0.05) (Figure 1).

#### 3.3.2. Chronic Zn, Cu, and Cd Stress on OCR of Sea Cucumber

**Figure 2:**
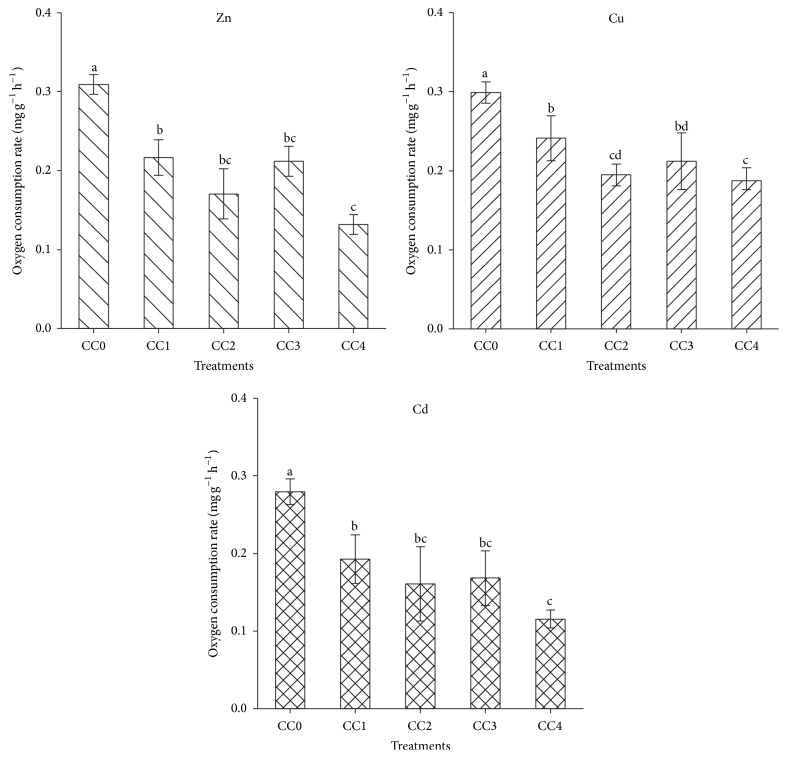
Levels of oxygen consumption rate of* Apostichopus japonicus* after 15 d chronic exposure to various Zn, Cu, and Cd concentrations. The bars are the respective standard deviations (*n* = 3), and different letters above the bars indicate significant differences (*P* < 0.05).

The OCRs in groups chronically exposed to metals were significantly lower than that in the control group (*P* < 0.05) (Figure 2). Significant difference for the OCR occurred only between CC4 and CC1 groups under Zn exposure (*P* < 0.05), while no significant differences were observed among the CC2, CC3, and CC4 groups. The same law was found in the Cd treated groups. In the Cu treated groups, the OCR of the highest concentration treated group was significantly lower than that in the lowest concentration treated group but not different from CC2.

#### 3.3.3. Effects of Chronic Zn, Cu, and Cd Exposure on HK and PK in Different Tissues of Sea Cucumber

**Figure 3:**
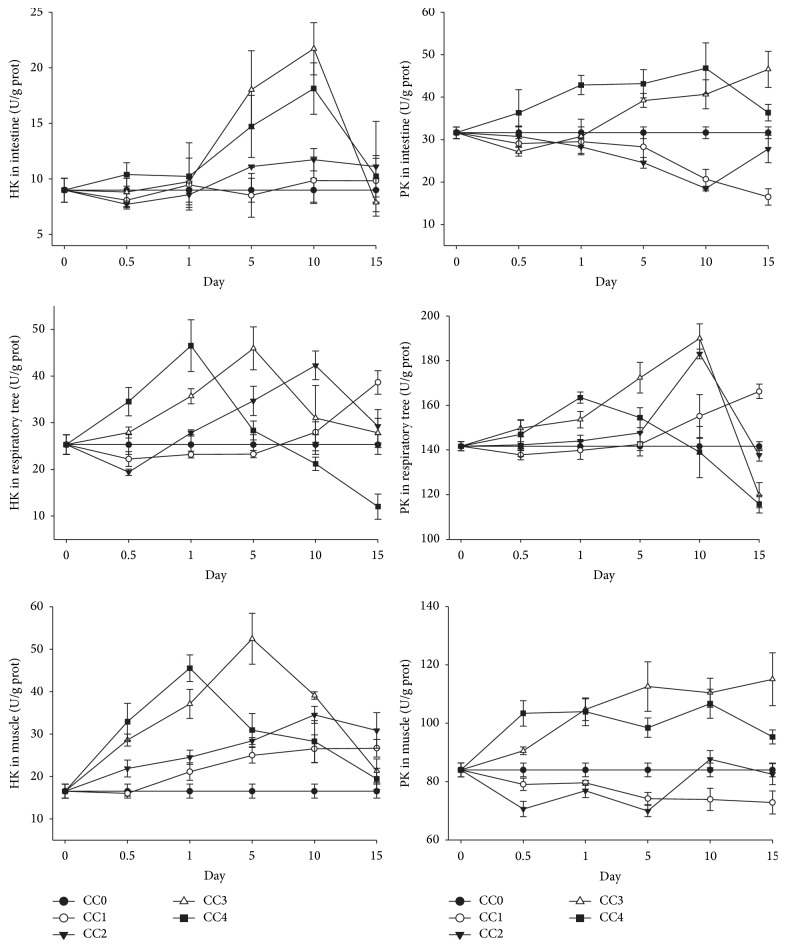
Levels of hexokinase (HK) and pyruvate kinase (PK) in intestine, respiratory tree, and muscle of* Apostichopus japonicus* during chronic Cu exposure. The bars are the respective standard deviations (*n* = 3).

In the nature seawater, the HK and PK activities in the respiratory tree were larger than those in the muscle followed by intestine (Figure 3). Under chronic low Cu exposure (0.003 mg L^−1^), the HK activity in the intestine did not change much and there was a slight trend of increase in the CC2 group (0.005 mg L^−1^) (Figure 3). However, the HK activity increased rapidly and reached the highest point on the 10th day of exposure and then decreased in the high concentration groups (CC3 and CC4). In the respiratory tree, which has a higher concentration of HK than the intestine under normal conditions, the change pattern of HK was different from the intestine. An increase of HK activity was observed in the low concentration group (CC1). In the other three groups, the HK activity increased at first and then decreased under chronic Cu exposure. The higher the exposure concentration was, the earlier the highest point of HK activity obtained. In the muscle, a slight increase of HK activity occurred in the two low concentration groups (CC1 and CC2), while in the two high concentration groups (CC3 and CC4), the HK activity increased rapidly and then decreased (Figure 3).

The PK in the respiratory tree responded similarly to the HK in this tissue (Figure 3). An increase of PK activity was observed in the low concentration group (CC1). In the other three groups, the PK activity increased at first and then decreased under chronic Cu exposure. The higher the exposure concentration was, the earlier the highest point of PK activity reached. A trend of increase was observed in both the intestine and muscle in the CC3 and CC4 groups. In the muscle, the PK in the low concentration groups (CC1 and CC2) fluctuated around the value in the nature seawater (Figure 3).

**Figure 4:**
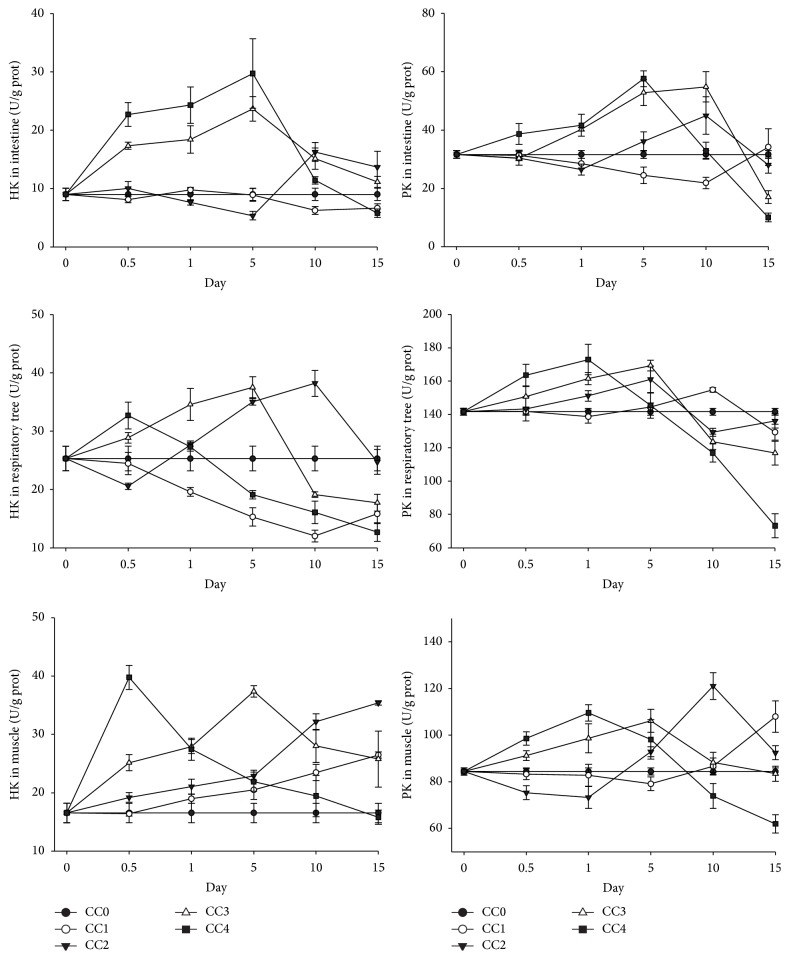
Levels of hexokinase (HK) and pyruvate kinase (PK) in intestine, respiratory tree, and muscle of* Apostichopus japonicus* during chronic Cd exposure. The bars are the respective standard deviations (*n* = 3).

Generally, the HK activity increased first and then decreased in all the three tissues under chronic Cd exposure in the CC2, CC3, and CC4 groups. The higher the exposure concentration was, the earlier the highest point of HK activity reached (Figure 4). For example, the highest concentrations of HK activity in the respiratory tree of the three groups, that is, CC4 (0.440 mg L^−1^), CC3 (0.088 mg L^−1^), and CC2 (0.044 mg L^−1^), were obtained on days 0.5, 5, and 10 of exposure, respectively. In the low concentration group (CC1), the HK activity decreased in the respiratory tree, increased in the muscle, and fluctuated around the value in the nature seawater in the intestine. The change pattern of PK was similar to HK and the PK activity increased first and then decreased in the CC2, CC3, and CC4 groups. The higher the exposure concentration was, the earlier the highest point of PK activity reached. The PK fluctuated around the value of control in the low concentration group (CC1) in the intestine and respiratory tree (Figure 4).

**Figure 5:**
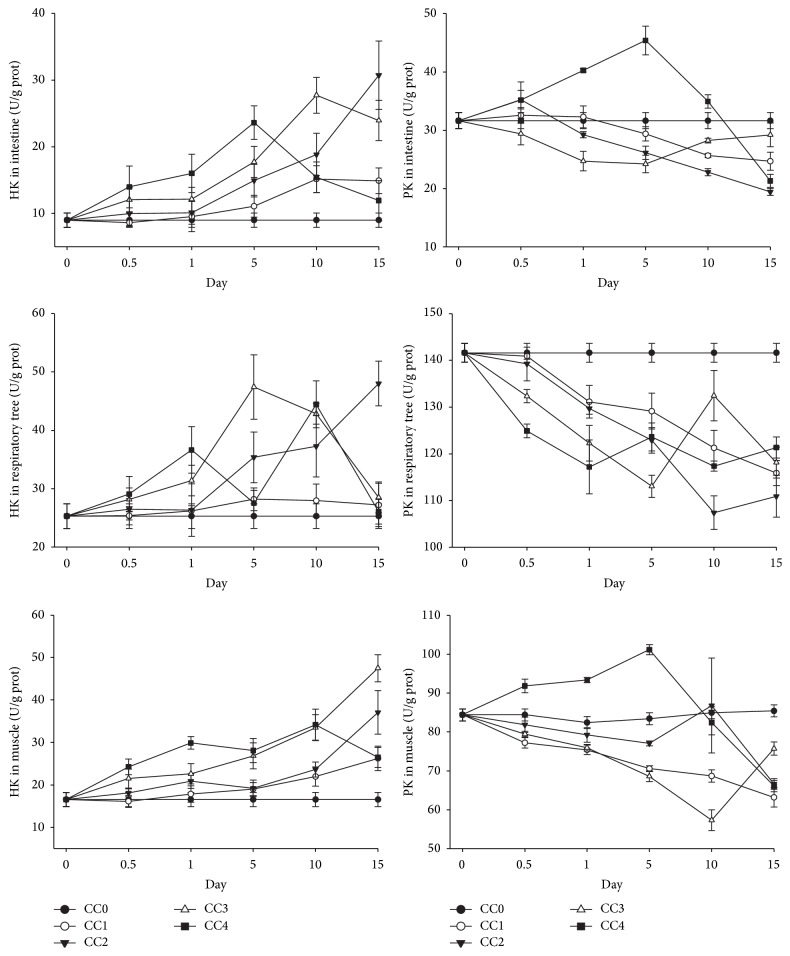
Levels of hexokinase (HK) and pyruvate kinase (PK) in intestine, respiratory tree, and muscle of* Apostichopus japonicus* during chronic Zn exposure. The bars are the respective standard deviations (*n* = 3).

Under chronic Zn exposure, in the CC2, CC3, and CC4 groups, the HK activity generally increased at first and then decreased in the respiratory tree and intestine (Figure 5). In the muscle, the HK activity increased in the four treatments and kept increasing in the CC1, CC2, and CC3 groups at the last sampling of this study. The PK activity increased and then decreased only in the intestine and muscle of the high concentration group, that is, CC4 group with a Zn concentration of 0.770. In the other treatments and tissues, the PK activity all decreased or fluctuated around control (Figure 5).

## 4. Discussion

When the sea cucumbers were acutely exposed to metals, severe mortality was observed in this study. However, mortality only occurred in groups treated with the highest concentration of heavy metals in the chronic toxicity test. This might be because of the acclimation response of the sea cucumber under chronic metal exposure. It has been reported that fish can be physiologically acclimated to chronic Zn exposure by reducing the branchial influx rate of Zn and restoring plasma calcium concentrations [19]. Cadmium is the most commonly found nonessential heavy metal in aquatic environments and it tends to bioaccumulate in living organisms. The metal, which could disrupt calcium absorption, can lead to acute hypocalcaemia and growth reduction, problematic reproduction, and impairments in development and behavior in aquatic species [4, 20]. As a key constituent of metabolic enzymes, Cu is an essential micronutrient for living organisms [4]. However, it can be toxic to aquatic organisms when exceeding normal levels. The toxic effect includes reduced growth rate, behavioral changes, and deformities in fish larvae [4]. An essential element for living organisms, Zn, is crucial to over 300 enzymes and other proteins and also a vital component of all their tissues and fluids of organs [21, 22]. However, it may have detrimental effects on the development and survival of many aquatic organisms when reaching a threshold [4]. The mechanism of its toxicity is similar to Cd, that is, disrupting calcium homeostasis through the induction of hypocalcaemia and disturbing acid-base balance [19].

The toxicity of certain metals is species-dependent. The 96 h LC50 value for Cd in sea cucumber is 1574 *μ*g L^−1^ (Table 5), while that for the rainbow trout, zebrafish, and perch is 19, 3822, and 8141 *μ*g L^−1^, respectively. This indicates that the sea cucumber is less sensitive to Cd toxicity than the rainbow trout, but more sensitive than the zebrafish and perch [23, 24]. The 96 h LC50 for Cu in sea cucumber is 133 *μ*g L^−1^, which is higher than rainbow trout, but lower than the zebrafish [24, 25]. The 96 h LC50 for Zn in sea cucumber is 2697 *μ*g L^−1^ and the sea cucumber is more tolerant to Zn toxicity than the rainbow trout, which has a 96 h LC50 of 869 *μ*g L^−1^ [26]. However, some other aquatic species, such as guppy, are extremely tolerant to Zn toxicity with a 96 h LC50 of 30826 *μ*g L^−1^ [27]. The sea cucumber's sensitivity to metals was metal-dependent. In this study, the sea cucumber is more sensitive to Cu than Cd. Similar result was reported for common carp and zebrafish [24]. However, some other species such as ridgetail white prawn (*Exopalaemon carinicauda*) are more sensitive to Cd than Cu [6], while other species, such as rainbow trout, are sensitive to both Cu and Cd [24]. The mechanisms leading to different sensitivities among species remain not clear and it may be related to differences in the regulation and affinity of metal uptake [24].

The change of oxygen consumption rate was reported in* Apostichopus japonicus* after evisceration or under fluctuant temperatures [16, 28]. Both Cd and Cu exposure could cause significant inhibition of OCR in* Exopalaemon carinicauda*. Similar results were also reported in* Farfantepenaeus paulensis *and* L. vannamei*, which was acutely exposed to Zn and Cd [5, 6, 8]. In this study, acute Cu and Cd stress resulted in the decrease of OCR in sea cucumber, which is consistent with previous reports. The decrease of OCR in* L. vannamei* was attributed to histopathological alterations in the gills after acute exposure to Cd and Zn [6, 29], while inhabitation of respiration by heavy metal in mussel has been attributed to mucus production because it reduces the efficiency of gaseous exchange [30]. However, the physiological mechanism for respiratory impairment in sea cucumber is not clear. Though OCRs were generally decreased when the aquatic animals were acutely exposed to heavy metals [5], it is interesting to find that the OCR in sea cucumber increased under acute low concentration Zn exposure (1.00 mg L^−1^). The exact reason for the surprising elevation of OCR in sea cucumber remains unknown. However, elevated OCR was observed in Green-lipped mussels after chronic exposure to raised Cd for one week and was interpreted as “an augmented expenditure of energy reserves characteristic of a stress compensation process” [7]. The primary respiratory organ in the sea cucumber is the respiratory tree and the animal could also obtain oxygen via cutaneous respiration [28]. The increase of OCR in the sea cucumber indicated elevated activity of the two parts.

In the nature seawater, the HK in the three tissues in sea cucumber followed: respiratory tree > muscle > intestine. Therefore, the metabolic responses of the three organs to heavy metal exposure were different and the respiratory tree was the most active metabolic place. The HK in the respiratory tree showed a trend of increase and then decrease in all the treatments except groups treated with the lowest concentration for all metals in chronic exposure test. And the higher the exposure concentration was, the earlier the highest point of HK activity obtained. HK is a key enzyme of glycolysis by converting glucose to glucose-6-phosphate [31]. The elevation of HK might be related to the glycolytic pathway to derive energy from glucose. The HK activity decreased when the time of duration of metal exposure increased indicating that the sea cucumber could not maintain the energy support from the glycolysis. The HK activity was reported to be affected by several factors such as salinity, dietary carbohydrate, and molt cycle in shrimp [14], while in sea cucumber, the variation of HK under metal exposure was not reported. In the present study, certain level of chronic metal stress seemed to promote glycolysis at initial phase. However, as the duration of metal stress lasted, the glycolysis was inhibited. The higher the metal concentration was, the early the inhibition happened.

The PK in the three tissues in sea cucumber also followed: respiratory tree > muscle > intestine. As a major site of acute hormone action, pyruvate kinase (PK) is also one of the key enzymes in control of the glycolytic pathways [32]. It catalyzes the transfer of a phosphate group from phosphoenolpyruvate (PEP) to ADP, yielding one molecule of pyruvate and one molecule of ATP (PEP + ADP → pyruvate + ATP) [33]. The specific activity of PK is affected by several factors such as different diets, while prolonged starvation could reduce the activity of PK in vertebrates. The mechanism of inhibition of PK is a hormone and metabolite-mediated mechanism in the regulation of the enzyme-gene expression [32]. Under chronically higher concentration Cd and Cu exposure, the PK in the respiratory tree increased at first and then decreased, while in the lowest groups (CC0), the PK slightly increased or fluctuated around the value in control. However, no elevation of PK levels was observed in the respiratory tree of sea cucumber exposed to chronic Zn stress indicating that sea cucumber might be more tolerant to Zn than the Cu and Cd.

## 5. Conclusion

We conducted chronic and acute toxicity test to evaluate the effect of one commonly found nonessential element (Cd) and two essential elements (Zn and Cu) on survival, specific growth rate, oxygen consumption rate, and activity of metabolic enzymes in sea cucumber. From this study, it demonstrated that chronic metal exposure could inhibit the growth of the sea cucumber and the specific growth rate of sea cucumber decreased with the increase of metal concentration. The 96 h LC50 values were calculated as 2.697, 0.133, 1.574 mg L^−1^ for Zn, Cu, and Cd, respectively, and the three metals were ranked in order of toxicity: Cu > Cd > Zn. The maximum allowable toxicant concentrations causing no harm to sea cucumber for the three metals are 0.135, 0.007, and 0.079 mg L^−1^ for Zn, Cu, and Cd, respectively. Under acute or chronic heavy metal stress, the sea cucumber has many physiological adaption mechanisms including decrease or increase of oxygen consumption rate and adjusted activity of metabolic enzymes.

## Figures and Tables

**Table 1:** The experimental design and nominal concentrations of Zn, Cu, and Cd in acute toxicity experiment test.

Cation	Concentration (mg L^−1^)						
	CA0	CA1	CA2	CA3	CA4	CA5	CA6
Zn	Control	1.00	1.43	2.05	2.94	4.21	6.00
Cu	Control	0.02	0.03	0.05	0.08	0.13	0.20
Cd	Control	0.50	0.66	0.87	1.15	1.51	2.00

Note: comparison was conducted among different concentration groups within each metal.

**Table 2:** The experimental design and nominal concentrations of Zn, Cu, and Cd in chronic toxicity test.

Cation	Concentration (mg L^−1^)				
	CC0	CC1	CC2	CC3	CC4
Zn	Control	0.040	0.070	0.150	0.770
Cu	Control	0.003	0.005	0.010	0.050
Cd	Control	0.022	0.044	0.088	0.440

Note: comparison was conducted among different concentration groups within each metal.

**Table 3:** Nominal and measured concentrations (mean ± SD, *n* = 3) of Zn, Cu, and Cd in test solutions.

Zn			Cu			Cd		
Nominal concentration (mg L^−1^)	Measured (mg L^−1^)	%	Nominal concentration (mg L^−1^)	Measured (mg L^−1^)	%	Nominal concentration (mg L^−1^)	Measured (mg L^−1^)	%
0.00	0.020 ± 0.001	—	0.00	0.003 ± 0.001	—	0.00	0.001 ± 0.000	—
0.04	0.036 ± 0.004	90.0	0.003	0.004 ± 0.000	133.3	0.022	0.026 ± 0.004	118.2
0.07	0.038 ± 0.001	54.3	0.005	0.006 ± 0.000	120.0	0.044	0.053 ± 0.001	120.5
0.15	0.121 ± 0.003	80.7	0.01	0.011 ± 0.000	110.0	0.088	0.097 ± 0.001	110.2
0.77	0.682 ± 0.005	88.6	0.02	0.019 ± 0.004	95.0	0.44	0.419 ± 0.001	95.2
1.00	0.932 ± 0.002	93.2	0.03	0.029 ± 0.004	96.7	0.50	0.437 ± 0.001	87.4
1.43	1.321 ± 0.080	92.4	0.05	0.043 ± 0.003	86.0	0.66	0.575 ± 0.003	87.1
2.05	1.980 ± 0.003	96.6	0.05	—	—	0.87	0.784 ± 0.002	90.1
2.94	2.820 ± 0.056	95.9	0.08	0.069 ± 0.007	86.3	1.15	1.030 ± 0.005	89.6
4.21	4.110 ± 0.073	97.6	0.13	0.123 ± 0.001	94.6	1.51	1.480 ± 0.001	98.0
6.00	5.230 ± 0.126	87.2	0.20	0.184 ± 0.001	92.0	2.00	1.820 ± 0.005	91.0

**Table 4:** Effects of acute Zn, Cu, and Cd stress on the survival rate of *Apostichopus japonicus*.

Cations	Concentration (mg L^−1^)	24 h Survival rate (%)	48 h Survival rate (%)	72 h Survival rate (%)	96 h Survival rate (%)
Zn	1.00	100	100	100	100
	1.43	100	100	100	100
	2.05	100	94.44	83.33	77.78
	2.94	94.44	83.33	66.67	33.33
	4.21	83.33	66.67	38.89	16.67
	6.00	66.67	38.89	11.11	0

Cu	0.02	100	100	100	100
	0.03	100	100	100	100
	0.05	100	100	100	100
	0.08	94.44	88.89	77.78	77.78
	0.13	88.89	77.78	55.56	50.00
	0.20	77.78	61.11	38.89	27.78

Cd	0.50	100	100	100	100
	0.66	100	100	100	100
	0.87	100	100	100	100
	1.15	94.44	88.89	77.78	66.67
	1.51	88.89	77.78	66.67	50.00
	2.00	33.33	72.22	55.56	38.89
	Control	100	100	100	100

**Table 5:** The regression equation and medium lethal concentration (LC50) of *Apostichopus japonicus* exposed to various Zn, Cu, and Cd concentrations calculated by probit analysis.

Cation	Time/h	Regression equation	*R* ^2^	LC50 (mg L^−1^)	MATC^*∗*^ (mg L^−1^)
Zn	24 h	*y* = 3.8072*x* + 1.6268	0.9970	7.691	
	48 h	*y* = 3.9949*x* + 2.1415	0.9968	5.200	
	72 h	*y* = 4.6655*x* + 2.4832	0.9873	3.467	
	96 h	*y* = 6.439*x* + 2.2450	0.9723	2.679	0.135

Cu	24 h	*y* = 2.1241*x* + 5.6999	0.9924	0.468	
	48 h	*y* = 2.3553*x* + 6.3485	0.9884	0.267	
	72 h	*y* = 2.6206*x* + 7.1363	0.9943	0.153	
	96 h	*y* = 3.3879*x* + 7.9710	0.9985	0.133	0.007

Cd	24 h	*y* = 2.7053*x* + 3.2471	0.9866	4.446	
	48 h	*y* = 2.6388*x* + 3.6658	0.9280	3.206	
	72 h	*y* = 2.5822*x* + 4.0910	0.9978	2.250	
	96 h	*y* = 2.9672*x* + 4.4159	0.9837	1.574	0.079

^*∗*^MATC (maximum allowable toxicant concentration).

**Table 6:** The survival rate and specific growth rate of *Apostichopus japonicus* exposed to various concentrations of Zn, Cu, and Cd for 15 days. Different letters indicate significant differences (*P* < 0.05).

Cations	Concentration (mg L^−1^)	Survival rate (%)	Initial wet weight (g)	Final wet weight (g)	Specific growth rate (% d^−1^)
Zn	Control	100	16.36 ± 0.25^ac^	21.32 ± 0.23^a^	21.64 ± 1.75^a^
	0.040	100	15.58 ± 0.15^b^	19.30 ± 0.25^b^	16.67 ± 1.29^b^
	0.070	100	15.74 ± 0.45^ab^	18.02 ± 0.61^c^	10.66 ± 1.45^c^
	0.150	100	16.12 ± 0.52^acb^	16.98 ± 0.60^d^	4.18 ± 0.38^d^
	0.770	58.30	16.48 ± 0.33^c^	13.07 ± 0.52^e^	−19.15 ± 1.73^e^

Cu	Control	100	16.36 ± 0.25^a^	21.32 ± 0.23^a^	21.64 ± 1.75^a^
	0.003	100	15.95 ± 0.04^bc^	19.05 ± 0.63^b^	14.11 ± 2.76^b^
	0.005	100	16.18 ± 0.11^ab^	18.30 ± 0.32^bc^	9.94 ± 2.33^c^
	0.010	100	15.75 ± 0.37^c^	17.72 ± 0.68^c^	9.26 ± 1.62^c^
	0.050	75.00	16.88 ± 0.09^d^	14.31 ± 0.31^d^	−13.94 ± 1.52^d^

Cd	Control	100	16.36 ± 0.25^ab^	21.32 ± 0.23^a^	21.64 ± 1.75^a^
	0.022	100	16.64 ± 0.26^b^	20.82 ± 0.64^a^	18.63 ± 1.55^a^
	0.044	100	16.67 ± 0.27^b^	19.27 ± 0.63^b^	12.09 ± 2.16^b^
	0.088	100	15.94 ± 0.2^a^	17.97 ± 0.41^c^	9.53 ± 2.65^b^
	0.440	83.30	16.05 ± 0.1^a^	13.23 ± 0.11^d^	−15.51 ± 1.26^c^

## Abbreviations

OCR: Oxygen consumption rate
HK: Hexokinase
PK: Pyruvate kinase
SGR: Specific growth rate
MATC: Maximum allowable toxicant concentration
AF: Application factor.
